# Human transbodies that interfere with the functions of Ebola virus VP35 protein in genome replication and transcription and innate immune antagonism

**DOI:** 10.1038/s41426-018-0031-3

**Published:** 2018-03-21

**Authors:** Watee Seesuay, Surasak Jittavisutthikul, Nawannaporn Sae-lim, Nitat Sookrung, Yuwaporn Sakolvaree, Wanpen Chaicumpa

**Affiliations:** 1 Center of Research Excellence on Therapeutic Proteins and Antibody Engineering, Department of Parasitology, Faculty of Medicine Siriraj Hospital, Mahidol University, Bangkok, 10700 Thailand; 2 Department of Research and Development, Faculty of Medicine Siriraj Hospital, Mahidol University, Bangkok, 10700 Thailand

## Abstract

Small molecular inhibitors and passive immunization against Ebola virus disease (EVD) have been tested in animal models, including rodents and non-human primates, as well as in clinical trials. Nevertheless, there is currently no Food and Drug Administration (FDA)-approved therapy, and alternative strategies must be pursued. The aim of this study was to produce cell-penetrable human single-chain antibodies (transbodies) that are able to interfere with the activities of interferon inhibitory domain (IID) of the VP35 protein, a multifunctional virulence factor of Ebola virus (EBOV). We speculated that effective VP35-IID-specific transbodies could inspire further studies to identify an alternative to conventional antibody therapies. Phage display technology was used to generate *Escherichia coli*-derived human single-chain antibodies (HuscFvs) that bind to IID. HuscFvs were linked to nona-arginine (R9) to make them cell penetrable. Transbodies of transformed *E. coli* clones 13 and 3, which were predicted to interact with first basic patch residues (R9-HuscFv13), central basic patch, and end-cap residues (R9-HuscFv3), effectively inhibited EBOV minigenome activity. Transbodies of *E. coli* clones 3 and 8 antagonized VP35-mediated interferon suppression in *VP35*-transduced cells. We postulate that these transbodies formed an interface contact with the IID central basic patch, end-cap, and/or residues that are important for IID multimeric formation for dsRNA binding. These transbodies should be evaluated further in vitro using authentic EBOV and in vivo in animal models of EVD before their therapeutic/prophylactic effectiveness is clinically evaluated.

## Introduction

VP35 protein is a multifunctional virulence factor for Ebola virus (EBOV) replication^1^. One of the functions of this protein is its polymerase co-factor activity that VP35 protein and other EBOV proteins, such as NP (nucleoprotein), VP30 (transcription factor), and L (RNA-dependent RNA polymerase) and viral RNA, form the viral replication complex^2^. VP35 interacts with NP for viral assembly^3^ and inhibits innate interferon (IFNα/β) production to facilitate host immune evasion^4–7^. The latter function is mediated by blocking the signaling pathways of RIG-1 and MDA5, either through sequestration of viral dsRNA^8,9^ or through direct contact with IKKε and TBK-1, which impairs the kinase interaction with downstream IRF-3/IRF-7 and IPS-1^10^ and leads to an absence of several integral antiviral factors that are normally generated via the autocrine and paracrine actions of IFNs, including PKR and 2΄5΄-OAS^11^.

The EBOV VP35 comprises 340 residues that contain the N-terminal oligomerization domain (NOD) (residues 1–220) and C-terminal portion that has IFN antagonistic activity (termed the IFN-inhibitory domain, IID)^12–14^. VP35 forms multimers via the coiled-coil motif within NOD, which facilitates the interferon-antagonist activity of IID^15^. Residues 20–48 bind NP and regulate NP assembly on viral RNA to facilitate RNA synthesis^16,17^. Residues 71–75 interact with host dynein LC8 to enhance viral RNA synthesis^18^. The IID consists of two subdomains: an N-terminal α-helical subdomain (residues 221–283) that contains four alpha helices (α1–α4) and a C-terminal β-sheet subdomain (residues 294–340) that comprises four antiparallel strands (β1–β4), a small α5, and a type II polyproline helix^13^. Within the IID, there are two conserved basic patches and additional border basic residues that play different but cooperative roles in EBOV replication^19^. The first basic patch (K222, R225, K248, and K251) is located in the N-terminal helical subdomain and is important for VP35 polymerase co-factor function as well as NP binding for the formation of nucleocapsid^19^. The second or central basic patch (R305, K309, R312, K339, R322, and K319) binds to dsRNA to inhibit the IFN signaling pathway^1,13^. Conserved F239 and I340 form a hydrophobic pocket called an “end-cap” that binds the blunt ends of dsRNA^20^. Several other basic residues (K282, R283, R298, and R300) located at the IID periphery contribute to VP35 polymerase co-factor function^20^. H240, which is located near the first basic patch, is critical for VP35 activities^19^.

There is currently no effective FDA approved agent or protocol for the treatment of Ebola virus disease (EVD). Thus, EBOV-infected patients can only be provided supportive care^21^. Several agents and treatment regimens have been tested in both animal models of EVD and in naturally infected humans^22–29^. Whether any are effective in naturally infected humans could not be determined because the outbreak was dwindling, numbers of treated cases were relatively small, and other treatments were also administered. Passive immunization using EVD-convalescent serum and KZ52 monoclonal antibody has had limited success^30–33^, while newer polyclonal approaches (such as ZMapp, MB-003 and other antibody cocktails) are able to reverse advanced EVD in non-human primates (NHPs) and/or effectively prevent morbidity and mortality in NHPs when administered as a post-exposure prophylactic^34–38^. It should be noted that the antibodies used in the passive immunization were mostly in an intact four-chain format that either inhibited cellular entry of the virus (antibody to GP1)^36,38^, neutralized secreted glycoprotein (sGP) for mitigation of pathogenicity^39^, inhibited the release of endosomal RNP into the cytoplasm (antibody to GP2)^32^ or caused antibody-dependent cell-mediated cytotoxicity^40^. The antibodies lacked the ability to interfere with the activities of intracytoplasmic proteins of the replicating virus.

It is typically difficult for hydrophilic and large molecules, such as conventional four-chain antibodies, to penetrate the mammalian plasma membrane^41^. Thus, the antibodies are unable to access intracellular targets. Recently, cell-penetrating peptides (CPPs) have been shown to deliver their molecularly linked biologically active molecules into living cells^42^. Typically, CPPs are positively charged, which facilitates electrostatic interactions with negatively charged cell-surface constituents. Nona-arginine (R9) is an example of a CPP that effectively delivers its cargo into the cytoplasm^42,43^. Given that VP35 is associated with several pivotal activities in the EBOV infectious cycle^1^, the aim of this study was to generate cell-penetrable human scFvs (R9-HuscFvs) or transbodies that can effectively interfere with VP35-IID functions. We speculate that effective VP35-IID-specific R9-HuscFvs could inspire further analyses to identify an alternative to conventional antibody therapies.

## Results

### Recombinant VP35 and IID

**Fig. 1:**
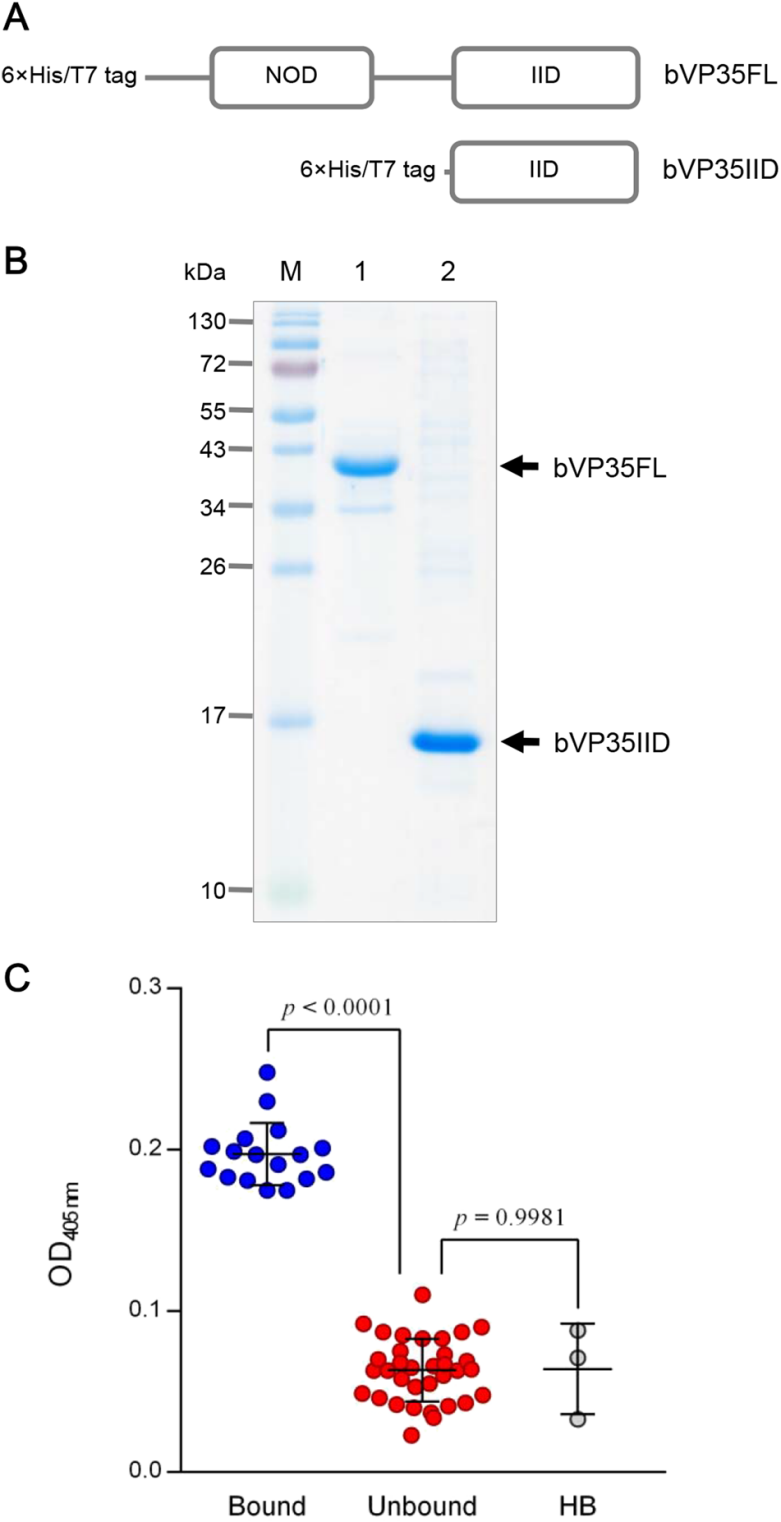
Productions of recombinant VP35 proteins and selection of VP35-bound HuscFvs. **a** Schematic representations of constructs of bacterially produced recombinant full-length EBOV VP35 (bVP35FL) and the C-terminal interferon inhibitory domain of VP35 (bVP35IID). **b** Recombinant bVP35FL and bVP35IID proteins purified from transformed *E. coli* clones. M, pre-stained protein ladder; lane 1, purified bVP35FL; and, lane 2, purified bVP35IID. Numbers at the left represent the protein molecular masses in kDa. **c** bVP358FL-bound HuscFv clones, as determined by indirect ELISA using purified bVP35FL as antigen. The bound group was selected from the OD_405nm_ signal above mean + 3SD of the background binding control (lysate of original *E. coli* HB2151; HB). Statistical significance was determined using one-way ANOVA and Tukey’s post hoc test. Supplementary Figure S1 provides details of the binding of individual clones to bVP35FL (test antigen) and BSA (control antigen)

Schematic diagrams of bacterially produced recombinant full-length EBOV VP35 (bVP35FL) and the C-terminal IID of VP35 (bVP35IID) are shown in Fig. 1a. SDS-PAGE-separated patterns of the purified recombinant proteins are shown in Fig. 1b.

### VP35-bound transbodies

Phage clones that bound to bVP35FL were selected from a HuscFv phage display library^44^ by bio-panning using 1 μg of bVP35FL as antigen. *E. coli* HB2151 infected with recombinant bVP35FL-bound phages were screened for *huscfv* sequences by PCR. Lysates of 17 and 34 *huscfv*-positive *E. coli* clones bound and did not bind to bVP35FL, respectively (Fig. 1c). Supplementary Figure S1 provides details of the binding of individual clones to VP35 (test antigen) and BSA (control antigen).

DNA coding for bVP35FL-bound HuscFvs of the 17 clones (No's. 3, 6, 7, 8, 10, 13, 15, 21, 23, 24, 25, 28, 29, 31, 33, 36, and 38) was classified into seven different types based on the deduced amino acid sequences: type 1 (clones 3 and 33); type 2 (clones 6, 7, 8, 10, 31, 36 and 38); type 3 (clones 13 and 21); type 4 (clone 15); type 5 (clone 23); type 6 (clones 24 and 29); and, type 7 (clones 25 and 28). Clones 3, 8, 13, 15, 23, 24, and 28 were selected as the representatives of individual types for further experiments.

**Fig. 2:**
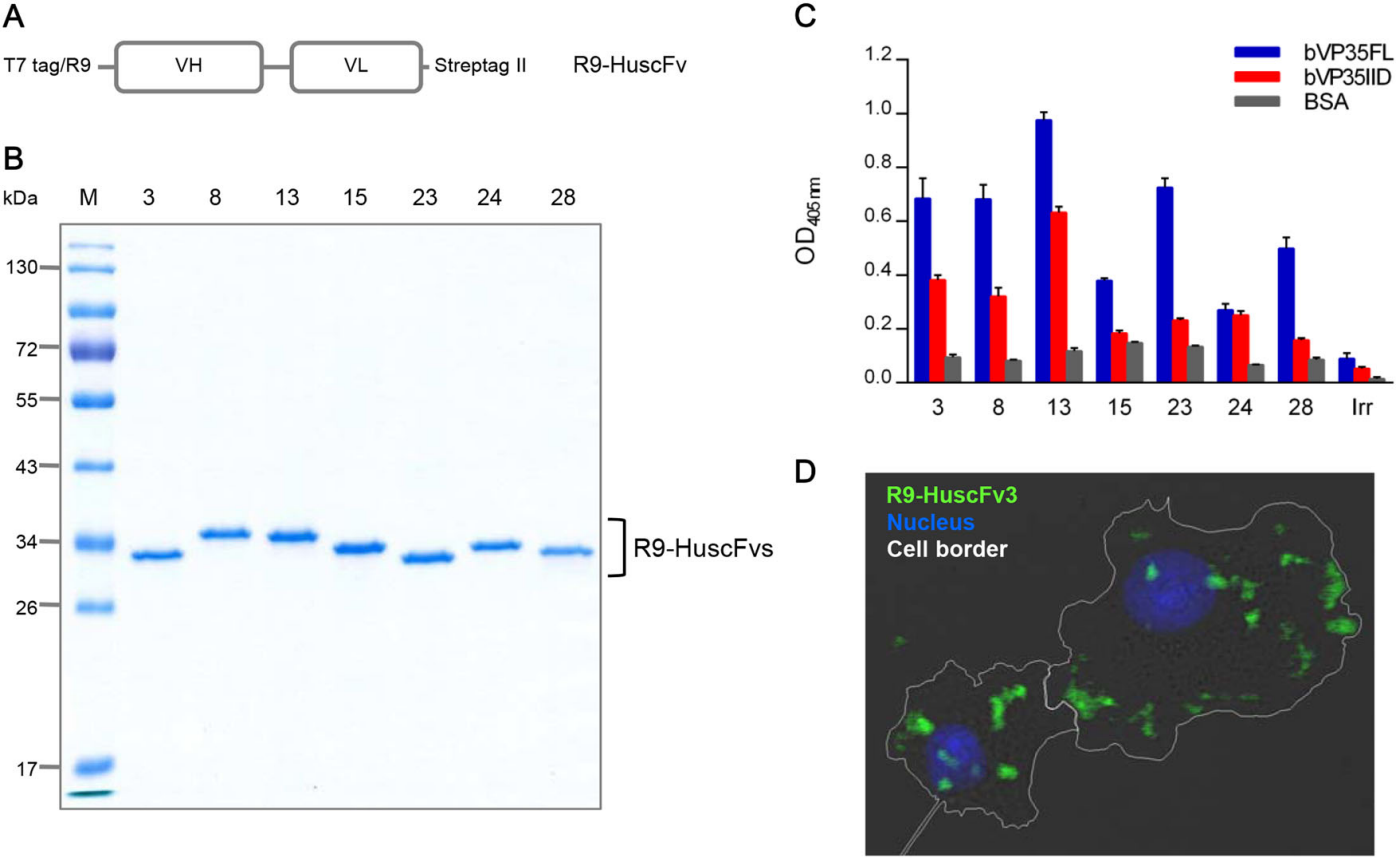
Antigen binding and cell entry ability of purified R9-HuscFvs. **a** Schematic representation of the construct for preparing cell-penetrable HuscFvs (R9-HuscFvs). **b** SDS-PAGE and CBB-stained R9-HuscFvs purified and refolded from transformed *E. coli* clones 3, 8, 13, 15, 23, 24, and 28. R9-HuscFvs had a molecular mass of ~34 kDa under reducing condition. **c** Binding activities of R9-HuscFvs to bVP35FL and bVP35IID compared to BSA (control antigen), as demonstrated by indirect ELISA. Positive binding to the tested antigens yielded an OD_405nm_ signal three times higher than to that of the control antigen. Supplementary Figure S2 shows the EC_50_ value derived from selected bVP35IID-bound R9-HuscFvs. **d** Intracellular localization of R9-HuscFv was revealed by confocal immunofluorescence microscopy. HepG2 cells were incubated with R9-HuscFv3 (representative of the R9-HuscFvs) for 3 h, and then the cells were fixed, permeabilized, and stained. Cell border, white line; R9-HuscFv, green; nuclei, blue

HuscFvs of *E. coli* clones 3, 8, 13, 15, 23, 24, and 28 were linked molecularly to R9, which is a CPP. Figure 2a shows a schematic diagram of the cell-penetrable HuscFv construct. Recombinant R9-HuscFvs were expressed as inclusion bodies (IBs) by the transformed *E. coli*. After IB purification and subsequent protein refolding, the purity of individual R9-HuscFvs (∼34 kDa) was checked by SDS-PAGE and CBB staining (Fig. 2b). The refolded R9-HuscFvs were tested for binding to bVP35FL and bVP35IID by indirect ELISA. All R9-HuscFvs retained their binding activity to bVP35FL; however, only the R9-HuscFvs of clones 3, 8, 13, and 24 bound to bVP35IID (ELISA signals for bVP35IID were more than three times the OD_405nm_ of that of the BSA control) (Fig. 2c). The EC_50_ values of R9-HuscFv3, 8, 13, and 24 bound to bVP35IID were 1.6, 1.12, 1.41, and 2.06 μM, respectively (Supplementary Figure S2). The R9-HuscFvs of these four clones were further tested for their cell entry ability. To achieve this goal, HepG2 cells were incubated with R9-HuscFvs from individual *E. coli* clones. Intracellular antibodies were probed with Chromeo 488-labeled anti-Strep tag II antibody and evaluated by confocal microscopy. The R9-HuscFvs of all clones were found to be cell penetrable, and they were located predominantly in the cytoplasm. Figure 2d depicts the intracellular localization of the R9-HuscFv3 as a representative model.

### Presumptive residues of VP35-IID that interact with HuscFvs

**Fig. 3:**
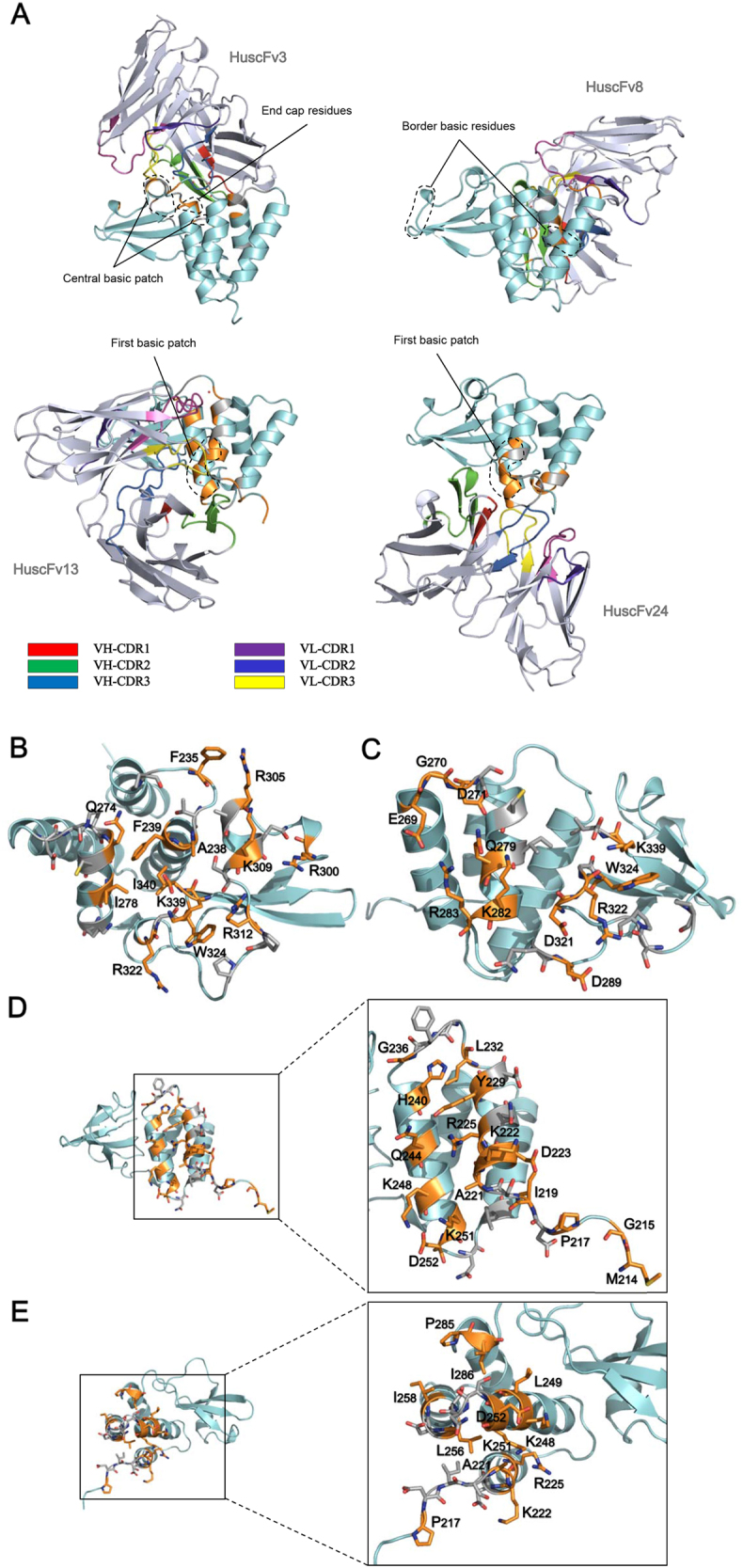
Computerized-generated structures of VP35-IID-HuscFv complexes and presumptive VP35-IID epitopes. **a** Overall structures of EBOV VP35-IID (PDB ID: 3FKE) (cyan) after complexing with HuscFvs (blue white) of *E. coli* clones 3, 8, 13, and 24 derived from molecular docking. Computer-generated animated images of VP35-IID that formed direct interface contact with antibodies are colored in orange, while the interfaces that fell within 5 Å thresholds of the van der Waals radii of the HuscFvs are colored in gray. **b–e** Contact interfaces between VP35-IID and HuscFv3, HuscFv8, HuscFv13, and HuscFv24. Residues of the IID that make contact with their respective HuscFvs are shown as sticks. For details, see Supplementary Table S1

The orientations of the complexes formed between VP35-IID and modeled HuscFvs are shown in Fig. 3a. The predicted presumptive residues on the contact interface of VP35-IID and individual HuscFvs are presented in Fig. 3b–e and Supplementary Table S1. According to the docking, the presumptive binding sites of HuscFv3 were at the spatially juxtaposed IID central basic patch interface (R305, K309, R312, R322, and K339), border basic residues (K282 and R300), and end-cap residues (F239 and I340). HuscFv8 was predicted to bind to the border basic residues opposite the IID first basic patch (K282 and R283), central basic patch (R322 and K339), and end-cap residue (I340). HuscFv13 interacted with the first basic patch interface (K222, R225, K248, and K251) as well as with H240. HuscFv24 formed a predictive interface with the IID helical subdomain and K222, R225, K248, and K251 of the first basic patch. Based on the important residues of IID that were predicted to form contact interfaces with HuscFvs (Supplementary Table S1), these antibodies were further tested to evaluate their ability to regulate VP35-IID activities.

### Binding of R9-HuscFvs to VP35 produced from mammalian cells (mVP35)

**Fig. 4:**
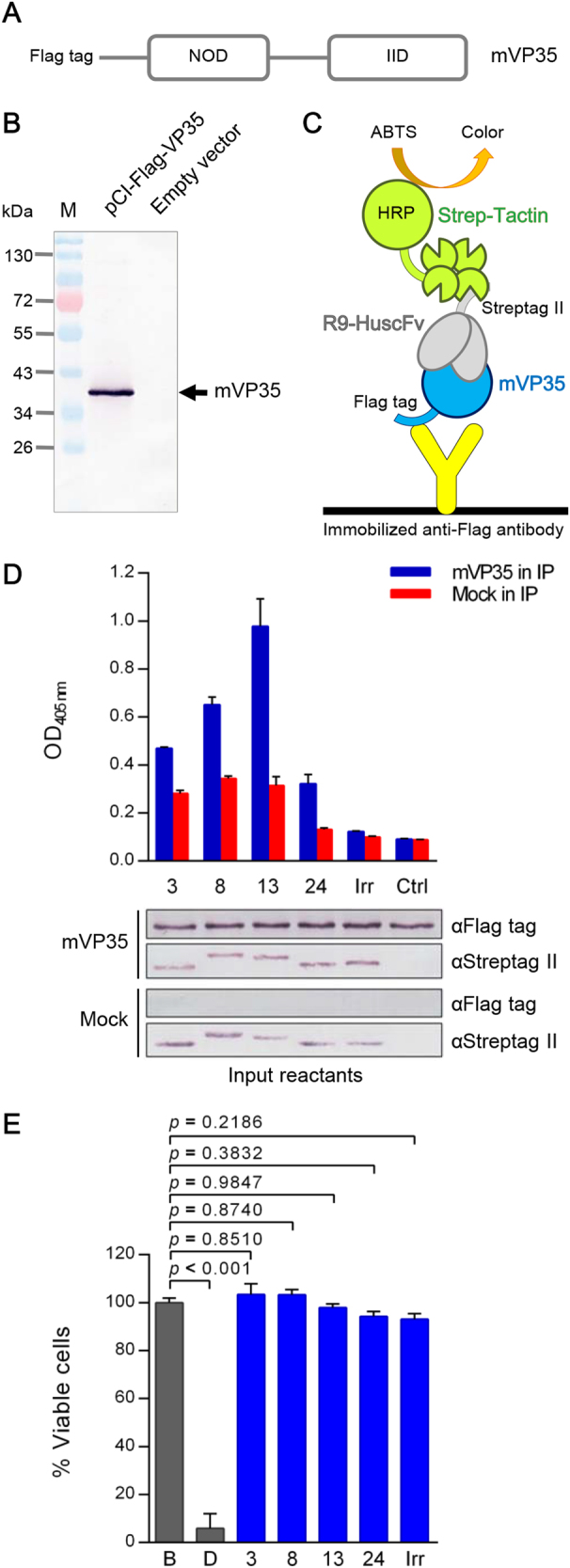
Interactions of the transbodies with VP35 and their biocompatibility. **a** Schematic representation of the VP35 construct for production in mammalian cells (mVP35). **b** mVP35 produced from COS-7 cells (∼40 kDa; arrow). COS-7 cells were transiently transfected with the VP35 construct. At 48-h post-transfection, the cells were lysed, and the presence of mVP35 in the clarified lysate was determined by Western blotting using anti-Flag antibody. The lysate of cells transfected with empty vector was used as a negative control. **c** Diagram of co-immunoprecipitation and ELISA for detecting the interaction between mVP35 and R9-HuscFvs. COS-7 cell lysate containing mVP35 was mixed with R9-HuscFvs, and individual mixtures were added to wells containing immobilized anti-Flag antibody. Captured complexes were detected using Strep-Tactin®-HRP conjugate and HRP substrate. **d** Upper, the OD_405nm_ of ELISA for demonstrating the interaction between mVP35 and R9-HuscFvs compared with mVP35 mixed with irrelevant R9-HuscFv (Irr) and the background binding control (Ctrl; mVP35 without R9-HuscFv). Lower, the input reactants of co-immunoprecipitation revealed by Western blotting are shown. **e** The biocompatibility of R9-HuscFvs with human hepatic cells shown as the percent viability of HepG2 cells after incubation with R9-HuscFvs for 24 h. The protease activity of dead cells was measured after addition of luminogenic substrate to wells containing treated cells, and the percent cell viability was calculated. Statistical significance was determined using one-way ANOVA and Tukey’s post hoc test

Figure 4a shows the DNA construct encoding VP35 with the N-terminal Flag-tag. The mVP35 found in the transfected COS-7 cell lysate is shown in Fig. 4b. Ten micrograms of cell lysate containing mVP35 was mixed with 3 μg of purified R9-HuscFvs, and the individual mixtures were added to the wells of a 96-well plate containing immobilized anti-Flag antibody. After incubation and washing, Strep-Tactin^®^-HRP conjugate was added to detect the R9-HuscFv-mVP35 complexes (Fig. 4c). The lower panel of Fig. 4d illustrates the input proteins in the co-immunoprecipitation assay. The ELISA results shown in upper panel of Fig. 4d demonstrate that all R9-HuscFvs bound to VP35 in the transfected COS-7 cell lysate.

### Effect of transbodies on hepatic cells

HepG2 cells were mixed with 100 μL of complete medium containing 25 μg/mL of individual VP35-bound-transbodies, control (irrelevant) transbody (Irr), or buffer (B) for 24 h. Cells with added digitonin (D) served as a maximal cytotoxic control. Cell death was measured using the luminescent assay. The numbers of viable cells for all treatments were then calculated and found to be more than 90% in all cases. The viability of transbody-treated cells did not differ from that of cells with only buffer added (100% viable cells) (*p* > 0.05), indicating that the transbodies were biocompatible with human hepatic cells (Fig. 4e).

### Efficacy of the transbodies in the suppression of EBOV minigenome activity

**Fig. 5:**
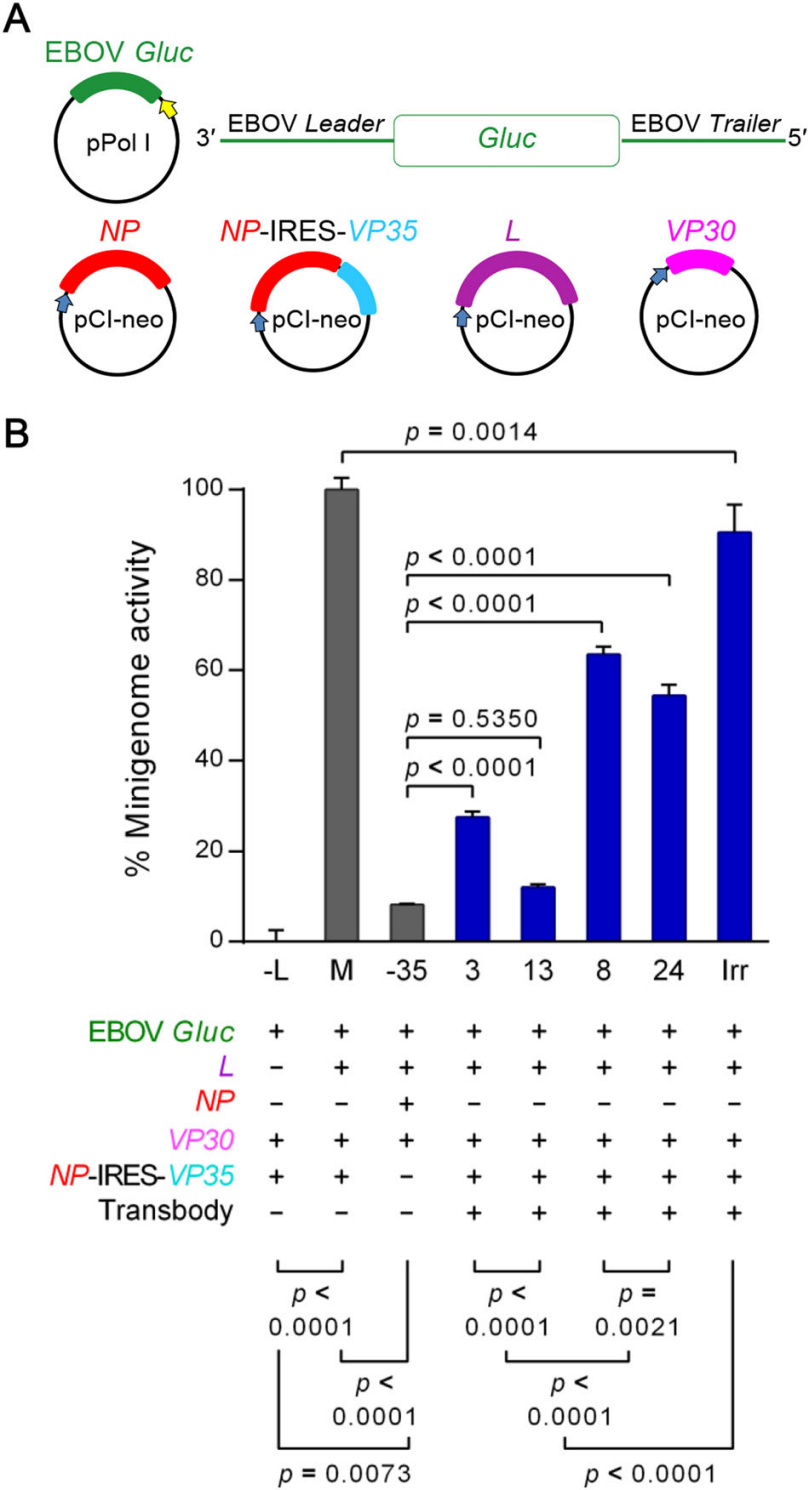
Transbody-mediated inhibition of EBOV minigenome activity. **a** Schematic diagrams of the plasmids used in the RNA polymerase I-driven EBOV minigenome system. EBOV-like reporter (Gluc) RNA was transcribed under the regulation of human Pol-I promoter and the Sal box transcription termination element. The viral protein expression cassettes were under the regulation of CMV I.E. enhancer/promoter and the SV40 late poly(A) signal element. **b** Percent EBOV minigenome activity of COS-7 cells after treatment with R9-HuscFvs. COS-7 cells transfected with EBOV minigenome were treated with R9-HuscFv3, R9-HuscFv8, R9-HuscFv13, R9-HuscFv24, and irrelevant R9-HuscFv (Irr). Minigenome activity was measured by detecting Gluc bioluminescent intensity. Controls included (M) COS-7 cells co-transfected with the minigenome without antibody treatment, (−L) COS-7 cells co-transfected with EBOV minigenome without *L* plasmid, and (−VP35) COS-7 cells co-transfected with the minigenome without *VP35* plasmid. Differences in percent minigenome activity were compared using one-way ANOVA and Tukey’s post hoc test

The plasmids used in the assay for determining the efficacy of transbodies for inhibiting EBOV minigenome activity are shown in Fig. 5a. In this experiment, COS-7 cells were co-transfected with a minigenome consisting of a plasmid cocktail, i.e., EBOV-*Gaussia* luciferase (Gluc)+NP−IRES−VP35+VP30+L. R9-HuscFv3, R9-HuscFv8, R9-HuscFv13, R9-HuscFv24, irrelevant R9-HuscFv (Irr), or medium alone (M, for positive minigenome activity control) that was then added to the cells. The negative minigenome activity controls were COS-7 cells co-transfected with Gluc+NP−IRES−VP35+VP30 (−L) and Gluc+L+NP+VP30 (−VP35). The preparations were maintained at 37 °C in a 5% CO_2_ incubator for 36 h. The activities of the minigenome, as indicated by Gluc intensities in cell culture wells, were monitored. As shown in Fig. 5b, the tested R9-HuscFvs caused a significant decrease in minigenome activity when compared to the positive minigenome activity (M) and Irr (*p < *0.05). The most effective transbody was R9-HuscFv13 [90% inhibition, which was not significantly different from the minigenome activity without the VP35 construct (NP-IRES-VP35)]. R9-HuscFv3, R9-HuscFv24, and R9-HuscFv8 inhibited VP35 genome activity by ∼70, ∼50, and ∼40%, respectively. The irrelevant transbody (Irr) also inhibited EBOV minigenome activity by ∼10% when compared to M (*p < *0.05).

### Transbodies mediated restoration of host innate gene expression in *VP35*-transduced cells

**Fig. 6:**
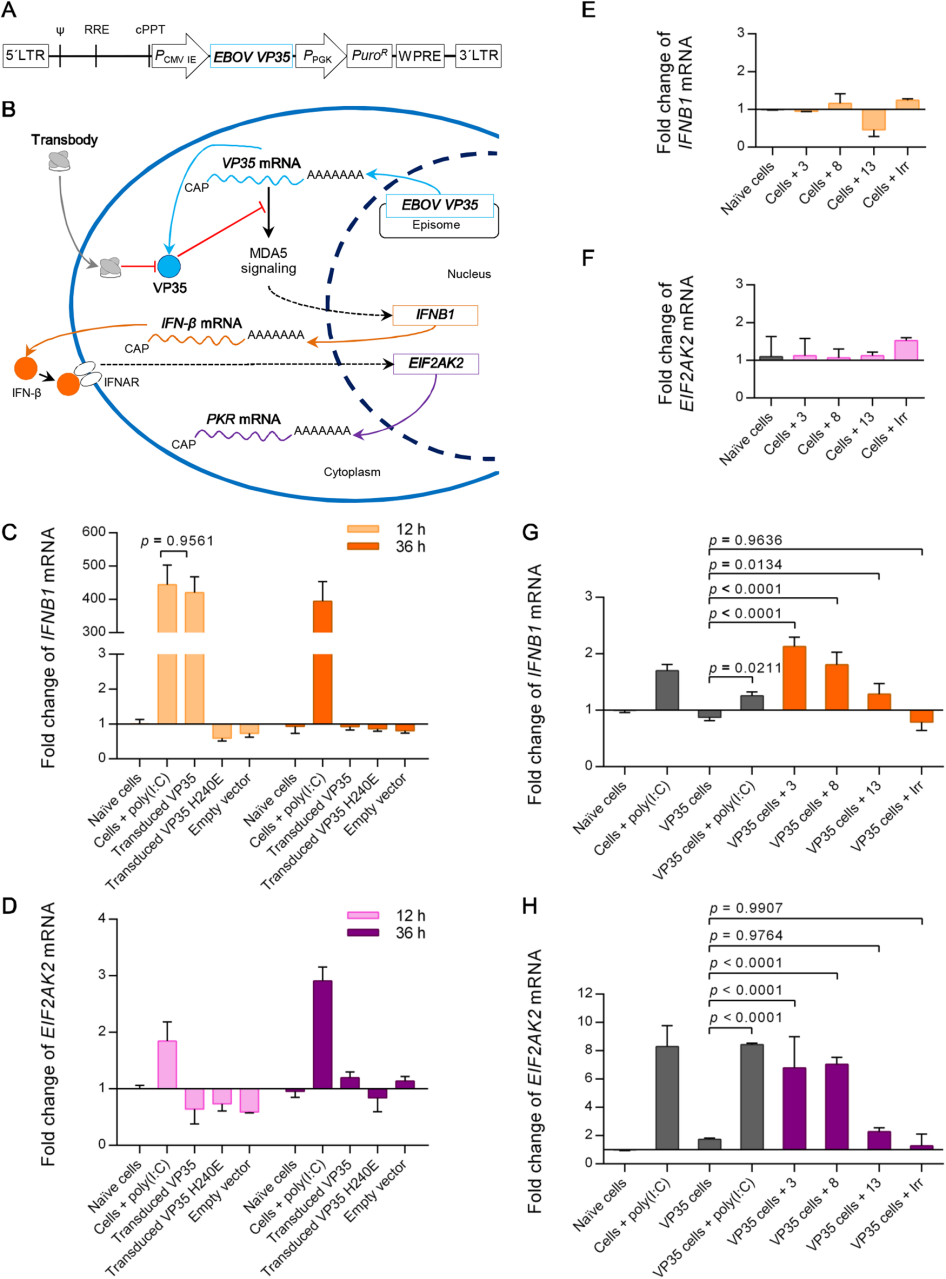
INF-β gene response in *VP35*-transduced cells after treatment with transbodies. **a** Schematic diagram of the EBOV VP35 expression cassette (pLVX-VP35) for HepG2 cell transduction. **b** Conceptual diagram illustrating how the transbodies rescued the IFN-β gene response of host cells from VP35 interferon antagonistic activity. After transduction with lentivector carrying EBOV VP35 gene cassette, transcription of the *EBOV-VP35* episome was initiated (cyan solid arrows). The ability of *VP35* mRNA to activate MDA5 signaling cascade was antagonized after VP35 production. Cytoplasmic VP35 bioactivity could be blocked by the VP35-targeting transbody (gray solid arrow), which resulted in *IFNB1* expression (orange solid arrows) by triggering transgene mRNA. As a consequence, the produced IFN-β induced interferon-stimulated genes (e.g., *EIF2AK2* (magenta solid arrow)). Dotted black arrows indicate signaling cascades. **c**, **d** Fold change of *IFNB1* (**c**) and *EIF2AK2* (**d**) mRNAs in cells after EBOV *VP35* transduction for 12 and 36 h compared to untreated control cells. Cells transfected with poly(I:C) served as positive stimulation, and cells transduced with EBOV *VP35H240E* and empty vector were transduction controls. Significant differences were determined by two-way ANOVA and the Šidák *t* test. **e**, **f** Expression of *IFNB1* (**e**) and *EIF2AK2* (**f**) mRNAs of untreated control HepG2 cells after incubation with transbodies for 12 h in comparison to the control cells in culture medium alone. **g**, **h** Fold change of *IFNB1* (**g**) and *EIF2AK2* (**h**) mRNAs in *VP35*-transduced cells after exposure to transbodies for 12 h compared with *VP35*-transduced cells. Controls included untreated cells with and without poly(I:C) transfection, *VP35*-transduced cells with and without poly(I:C) transfection, and *VP35*-transduced cells treated with irrelevant transbody (Irr). Statistically significant differences were determined using one-way ANOVA and Dunnett’s post hoc test

Figure 6a shows a schematic diagram of the VP35 expression cassette used for HepG2 transduction. The conceptual diagram regarding how EBOV VP35 mediates inhibition of the innate interferon signaling pathway and the expected VP35 antagonistic activity of the transbodies is illustrated in Fig. 6b. The ability of extra-chromosomal transgene expression to trigger the interferon response circuit^45^ was investigated. Expression levels of *IFNB1* and *EIF2AK2* (encode IFN-β and PKR, respectively), which are the integral antiviral genes in the interferon signaling pathway in HepG2 cells transduced with the VP35 gene cassette, was determined at 12-h and 36-h post-transduction. Expression of the *IFNB1* and *EIF2AK2* genes was assessed by quantitative reverse transcription-polymerase chain reaction (qRT-PCR). Controls were normal cells, cells transfected with poly(I:C), and cells transduced with VP35H240E gene cassette and empty vector. At 12 h, cells transduced with *VP35* gene and poly(I:C) showed comparable upregulation of *IFNB1* (∼400-fold) compared with normal cells (Fig. 6c). At 36 h, the expression level of *IFNB1* in cells with the VP35 transgene returned to the level in normal cells, while the expression level of *IFNB1* in cells treated with poly(I:C) was sustained at 36 h. In contrast, the *EIF2AK2* expression patterns were similar to those observed in cells transduced with empty vector (Fig. 6d). Cells stimulated with poly(I:C) showed upregulated *EIF2AK2* at both time points. It is noteworthy that while the *VP35* construct could induce *IFNB1* expression, the *VP35H240E* construct could not. At the protein level, it has been previously shown that VP35H240E has lost its co-polymerase and IFN antagonistic activities^19^.

The effects of R9-HuscFvs on the innate immune response genes of HepG2 cells were investigated. VP35-specific R9-HuscFvs and irrelevant R9-HuscFv were added to cells in culture wells. After 12 h, the expression levels of *IFNB1* and *EIF2AK2* were determined. R9-HuscFvs did not cause a significant upregulation of *IFNB1* and *EIF2AK2* compared with the untreated control cells (*p* > 0.05) (Fig. 6e, f).

To investigate the effect of VP35-specific R9-HuscFvs on the interferon antagonistic activity of VP35, HepG2 cells maintained in complete medium were transduced with lentivector carrying the EBOV VP35 gene cassette (Fig. 6a). At 24 h post-transduction, the spent culture medium in each well was replaced with fresh medium containing VP35-specific R9-HuscFvs. Controls included untreated cells, cells stimulated with poly(I:C), *VP35-*transduced cells without any treatment, *VP35-*transduced cells stimulated with poly(I:C), and *VP35*-transduced cells treated with irrelevant R9-HuscFv. The treated cells were incubated for 12 h, and the expression levels of *IFNB1* and *EIF2AK2* were determined. R9-HuscFv3 and R9-HuscFv8 mediated the upregulation of both innate immune response genes compared with *VP35-*transduced cells in medium alone (*p < *0.0001) (Fig. 6g, h). *VP35-*transduced cells treated with R9-HuscFv13 and cells transduced with added poly(I:C) showed an equivalent and modest upregulation of *IFNB1* activity when compared with *VP35-*transduced cells alone (*p < *0.05) (Fig. 6g). Irrelevant R9-HuscFv did not cause any change in *IFNB1* expression. Regarding *EIF1AK2* (Fig. 6h), R9-HuscFv3 and R9-HuscFv8 upregulated gene expression by ∼7-fold, which was comparable to the observed gene expression in poly(I:C)-treated *VP35*-transduced cells. The effectiveness of R9-HuscFv13 on innate gene expression did not differ from that of irrelevant R9-HuscFv and untreated *VP35-*transduced cells.

## Discussion

An effective therapeutic agent and protocol for EVD are needed. Passive immunization and previously tested immunotherapy could not interfere with the replication of virus that has gained entry into host cells. The strategy being proposed in this study is to use small antibody fragments that can penetrate the cell and gain access to pivotal proteins of the replicating virus. The VP35 protein was selected not only to inhibit viral replication, translation, and RNP assembly but also to restore host immunity and reduce immunopathophysiology caused by EBOV^46,47^.

HuscFvs to VP35 were derived from human immunoglobulin genes, and therefore they should not be immunogenic in humans. Each molecule of the HuscFv contains six complementarity-determining regions (CDRs), and each CDR comprises several residues that can cooperate in capturing the target, which in this study consisted of several highly conserved critical residues pivotal for the function of VP35-IID^12^. Two recent studies, one employing V_H_H as cross-linkers of highly conserved regions of filoviral nucleoprotein to inhibit viral replication^48^ and the other evaluating the anti-MARV V_H_H epitopes as highly conserved^49^, antecede that targeting vital conserved regions of intracellular viral antigens may be less prone to selection of escape mutants. Escape mutants of GP were identified in EBOV-infected Rhesus monkeys treated with antibody cocktails (MB-003)^50^. Thus, a novel approach for antiviral therapeutics should be directed towards the conserved regions of the viral proteins, which are exemplified by residues in the first, border, and central basic patches of VP35 in this study (mutations at those residues render the protein inactive or functionally impaired^19,20^). Given that the function of the VP35 N-terminal domain is still elusive in contrast to the well-known structure and activities of the C-terminal domain (IID), we selected only the HuscFvs (clones 3, 8, 13, and 24) that recognized VP35-IID for further functional assays. HuscFvs were converted into a cell-penetrable format by linking them to a CPP, R9. R9-HuscFvs readily entered the cells and did not cause cytotoxicity to mammalian cells, which indicated their biocompatibility.

Basic residues of the IID first basic patch (R225, K248, and K251) interacted with the viral nucleoprotein (NP) for RNP assembly; this interaction is also essential for viral polymerase activity^19^. R225A, K248A, and K251A show markedly reduced NP-binding activity and a complete absence of polymerase co-factor activity^19^. The other basic amino acids located at the border of the IID (K282, R283, R298, and R300) are also important for VP35 polymerase co-factor function. Alanine mutants of these residues have diminished polymerase co-factor activity, although they retain their NP-binding capacity^19^. The polymerase co-factor activity was determined by the basic charge of the amino acids and not their identity^19^. A computerized simulation indicated that R9-HuscFvs of all clones formed contacts with residues that are important for VP35 polymerase co-factor function and/or interactions with NP, either the border basic residue(s) and/or the first basic patch residues. Therefore, they were tested for their ability to inhibit EBOV minigenome activity.

In this study, the EBOV minigenome activity assay was used to determine the antagonistic role of transbodies toward VP35-IID during EBOV transcription and replication. The system described by Jasenosky et al.^51^ consisting of the RNA polymerase I-driven minigenome and bicistronic NP and VP35 was used, which is more robust than the conventional minigenome system^51,52^. R9-HuscFv13, which was predicted to dock on K222, R225, K248, and K251 of the first basic patch and on the H240 end-cap, was the most effective transbody for inhibiting minigenome activity, which is consistent with a previous finding showing that alanine mutation of R225, K248, and K251 not only leads to a loss of polymerase co-factor function but also a reduced NP interaction^19^. The H240E mutant was found to lack both IFNβ-antagonistic and minigenome activities^19^, which indicated the important dual role of this amino acid in IID. The K222A mutation, however, retained activity comparable to that of wild type^19^. R9-HuscFv3, which was predicted to form contacts with K282 and R300 on the IID border, was the second most effective transbody for inhibiting EBOV minigenome activity, consistent with a previous observation that individual mutations of these two residues to alanine result in diminution of polymerase co-factor activity^19^. Presumptive R9-HuscFv24-mediated contact with K222, K225, K248, and K251 was similar to that of HuscFv13; however, this transbody was not as effective as R9-HuscFv13 for reducing EBOV minigenome activity, potentially due to a lack of H240 functional inhibition or different orientations of the antibody–IID interactions, as shown by molecular docking. The least effective R9-HuscFv8 was predicted to bind to the border basic residues K282 and R283. Although mutations of residues in the border basic patch have been shown to interfere with polymerase co-factor function^19^, R9-HuscFv8-inhibited minigenome activity by only ~40%, which indicated that these residues might be less important for genome activity. The mutations that led to discontinued co-polymerase activity observed previously^19^ may have been due to structural alterations of the protein.

VP35-IID in multimeric form binds efficiently to different viral RNA ligands, which results in inhibition of innate IFN signaling^20^. Important amino acids involved in the IID intermolecular interaction are located in the central basic patch, including R312 and R322 of the first monomer, which form hydrogen bonds to residues of the second monomer (G270, D271, and E269 and Q262 and E269, respectively)^20^. The interaction of IID with dsRNA was mediated by P233, T237, F239, S272, N274, C275, I278, A306, S310, and I340 of the first and third monomer via van der Waals and water forces, while R312 and R322 of the second and fourth IID molecules interacted with the phosphodiester backbone of the dsRNA^20^. In this study, computerized simulations suggested that HuscFv3, HuscFv8, and HuscFv13 formed an interface contact via van der Waals forces, hydrophobic forces, π effect, and/or hydrogen interactions with several residues of the IID (Supplementary Table S1). The end-capped dsRNA of these residues directly contacted the dsRNA and/or formed a multimeric complex during dsRNA binding^20^. Therefore, the R9-HuscFvs of the three clones were tested for their ability to inhibit VP35-IID IFN antagonistic activity and to restore innate immune response gene expression in HepG2 cells that had been transduced with a lentivector carrying the EBOV-VP35 gene. VP35-transduced cells treated with R9-HuscFv3 and R9-HuscFv8 showed an upregulation of *IFNB1* and *EIF2AK2* compared to the expression of *IFNB1* and *EIF2AK2* in VP35-transduction control and irrelevant R9-HuscFv-treated VP35-transduced cells. The predicted IID residues specific by HuscFv3 included the end-cap motif (F235, F239, and I340), other residues that have been known to contact dsRNA (P233, T237, Q274, C275, I278, A306, and S310), residues of the central basic patch that are important for interferon inhibitory activity (R305, K309, R312, R322, and K339), and D271 making up the IID multimeric contact^20^. Therefore, it is plausible that host innate immunity rescued by R9-HuscFv3 occurred via obstruction or suppression of these critical IID residues. R9-HuscFv8 showed comparable effectiveness to R9-HuscFv3 relative to host innate immunity restoration, which could be due to binding of the transbody to R322 and K339 of the central basic patch, which is important for the interaction and recognition of dsRNA^53^. In addition, R9-HuscFv8 was predicted to bind to I340 of the end cap and to S272, C275, I278, and Q279, which should disrupt dsRNA binding. Moreover, relative to that prediction, this transbody also bound to residues (E269, Q270, and D271), which form multimers during dsRNA binding of VP35. From computerized intermolecular docking analysis, R9-HuscFv13 docked on F235 for end-cap recognition and on H240 near the first basic patch. A previous study found that IFN antagonistic activity was not affected by the VP35-F235A mutant, while H240E caused increased IFN-β promoter activity^19,20^. However, R9-HuscFv13 only modestly rescued *IFNB1*, which did not result in a significant upregulation of the *PKR* gene compared with the upregulation observed in VP35-transduced cells. H240 may not be directly involved in interferon antagonistic activity. The structural instability of or configuration change in the H240E mutant may render the IID unable to retain its functions. The observed lowest level of effectiveness of R9-HuscFv13 for restoring the innate immune response might be because this transbody did not bind to the critical residues involved in dsRNA contact, such as those in the central basic patch or the border basic residues.

In conclusion, VP35-IID-specific transbodies are able to inhibit protein functions associated with the EBOV replication cycle, including polymerase co-factor activity and host IFN-antagonism. Further experiments are needed to elucidate the actual interaction between the antibodies and VP35-IID. These transbodies should be further tested using authentic EBOV before they can be considered a promising alternative to existing treatment approaches.

## Materials and methods

### Cell cultures

COS-7 cells (African green monkey kidney cell line), HepG2 cells (human hepatoma cell line), and Lenti-X 293 T cells were grown in complete medium (Dulbecco’s Modified Eagle’s Medium) (Gibco, Thermo Fisher Scientific, Waltham, MA, USA) supplemented with 10% heat-inactivated fetal bovine serum (FBS) (HyClone, GE Healthcare Life Sciences, Marlborough, MA, USA), 100 units/mL penicillin and 100 μg/mL streptomycin, and 2 mM l-alanine-l-glutamine dipeptide (Gibco) at 37 °C in a 5% CO_2_ atmosphere.

### Production of bacterially produced recombinant full-length EBOV VP35 and the C-terminal interferon inhibitory domain of VP35

The VP35 consensus sequence was obtained by multiple sequence alignments of Zaire EBOV VP35 from the universal protein resource^54^. Optimized DNA coding for VP35 (accession MF801599) was synthesized (GenScript, Picataway, NJ, USA) and used as a template for preparing bVP35FL and VP35-IID (residues A221-I340; bVP35IID) by PCR. DNA fragments were cloned into pLATE52 (Thermo Fisher Scientific) using the LIC technique. The respective recombinant plasmids were transformed into *E. coli* BL21 (DE3). Appropriate colonies of transformed *E. coli* were grown in LB broth containing 100 μg/mL ampicillin (LB-A) and 1 mM IPTG (Thermo Fisher Scientific). IBs containing bVP35FL and bVP35IID were purified, and both proteins were refolded (Supplementary Method S1).

### Phage bio-panning

Phage clones that bound to bVP35FL were selected from a HuscFv phage display library^44^ by bio-panning. One microgram of bVP35FL in 100 μL of 0.2 M sodium carbonate-bicarbonate buffer, pH 9.4 was coated into a well of an EIA/RIA strip (Corning, NY, USA). After incubation, the antigen-coated well was blocked with protein-free blocking buffer, followed by addition and incubation of the phage library with the immobilized antigen. After removing antigen-unbound phages by washing, log-phase *E. coli* HB2151 were added, and phage infection was permitted for 10 min. Bacteria were spread onto LB-A agar plates and incubated at 37 °C overnight. Phagemid-transformed *E. coli* colonies that appeared on the plates were screened for HuscFv genes (*huscfvs)* by direct colony PCR^44^. The *huscfv-*positive *E. coli* clones were grown in 2YT broth containing 100 μg/mL ampicillin (2YT-A) and 2% w/v glucose at 37 °C for 3 h. The bacterial pellet was suspended in 2YT-A medium containing 0.2 mM IPTG and incubated at 30 °C with shaking for 5 h. Cells were harvested from individual cultures, suspended in PBS and sonicated. Binding of HuscFvs in soluble *E. coli* fractions to bVP35FL was tested by indirect ELISA (Supplementary Method S2). Phagemid DNA from *E. coli* clones that produced rVP35FL-bound HuscFvs were subjected to nucleotide sequencing, and then the CDRs and immunoglobulin framework regions of all sequences were identified using the integrative database of germ-line variable genes (VBASE2, http://www.vbase2.org)^55^.

### Production of R9-HuscFvs (transbodies) to VP35

An optimized DNA construct of R9-HuscFv-Strep tag II with an additional stop codon was synthesized and cloned into pET24a^+^ (GenScript). The DNA was introduced into *E. coli* BL21 (DE3). For production of R9-HuscFvs, the transformed *E. coli* BL21 (DE3) clones were cultured in LB broth containing 30 μg/mL kanamycin at 37 °C with aeration overnight. An aliquot of 25 μL of the overnight culture were inoculated into 250 mL of fresh auto-induction KPM medium containing 50 μg/mL of kanamycin. The cultures were maintained at 30 °C with shaking at 250 rpm overnight. Bacterial cells were harvested by centrifugation (4000 × *g* at 4 °C for 20 min). The antibodies were purified as described in Supplementary Method S1 before use in subsequent experiments. The purified and refolded recombinant R9-HuscFvs were verified by SDS-PAGE and Coomassie Brilliant Blue G-250 (CBB) staining. Their binding activity and half maximal effective concentrations (EC_50_) were determined (Supplementary Method S2).

### Confocal immunofluorescence microscopy

HepG2 cells (6 × 10^4^ cells) were placed in an 8-chamber cell imaging cover-glass (Eppendorf, Hamburg, Germany) and kept at 37 °C in a 5% CO_2_ incubator overnight. The culture medium was removed, and the cells were replenished with 0.5 mL fresh complete medium containing 25 µg/mL of individual R9-HuscFvs and then further incubated for 3 h. The cells were gently rinsed with Dulbecco’s PBS (DPBS) (Gibco), fixed with a 1:2 dilution of IC fixation buffer (eBioscience, Thermo Fisher Scientific) in DPBS at 25 °C for 1 h, washed with PBS, permeabilized using 0.1% v/v Triton X-100 in PBS at 37 °C for 30 min, and blocked with blocking solution (1% w/v BSA, 22.52 mg/mL glycine, and 0.1% v/v Tween-20 in PBS) at 25 °C for 1 h. After blocking, the cells were washed with PBS and stained with StrepMAB ImmoChromeo 488-conjugated antibody (IBA Life Sciences GmbH) and 1 µg/mL Hoechst 33342 (Biotium, Fremont, CA, USA) in blocking solution at 4 °C overnight. After staining, the cells were washed with PBS, mounted with 50% w/v glycerol in PBS, and observed under a confocal microscope (Carl Zeiss Laser Scanning System LSM 510, Carl Zeiss Microscopy GmbH). Images were processed using the Zeiss LSM Image Browser (version 3.2.0.115).

### Computerized simulation

The crystal structure of the VP35-IID (PDB ID: 3FKE) was obtained from the Protein Data Bank (RCSB PDB). Amino acid sequences of HuscFvs were subjected to homology modeling by iterative threading assembly refinement (I-TASSER)^56^. To improve the local geometric and physical quality of the predicted 3D structure, I-TASSER predicted models were refined using high-resolution protein structure refinement and fragment-guided molecular dynamics simulation. The VP35-IID structure and the modeled HuscFvs were subjected to antibody-protein docking (ClusPro)^57^.The largest cluster size that showed all of the HuscFv-CDRs within the 5 Å distance of the VP35-IID was selected. For interaction analysis, the antibody-protein complexes in the NAMD simulated environment were adopted^58^. Pymol software (version 1.3r1 edu) (Schrödinger, New York, NY, USA) was used to visualize the modeled complex. Residues of VP35-IID with side chains that fell within the threshold of 5 Å of HuscFv radii (van der Waals radii of interacting atoms and water) were defined as making interface contact with the antibody.

### Co-immunoprecipitation assay

The *Nhe*I*-Flag tag-MCS-Myc tag-Not*I DNA fragment (Integrated DNA Technologies, Coralville, IA, USA) was used to replace the original multiple-cloning sites of the pCI-neo vector (Promega, Fitchburg, WI, USA). The recombinant vector was designated pCI-Flag/Myc. Codon-optimized DNA coding for EBOV VP35 (accession no. MF801600) for mammalian expression was used as a template for preparing a Flag-VP35-expressing construct. EBOV VP35-coding DNA was amplified and cloned into pCI-Flag/Myc via the *Eco*RI and *Not*I restriction sites. The recombinant plasmid and the empty vector were propagated separately in *E. coli* JM110, and they were isolated using a Presto™ Endotoxin Free Mini Plasmid Kit (Geneaid Biotech, New Taipai City, Taiwan). COS-7 cells were placed in wells of a 6-well cell culture plate (Corning) (5 × 10^5^ cells/well) and incubated overnight. Five micrograms of each plasmid preparation was transfected separately into cells using Xfect Single Shots (Midi) Transfection Reagent (Takara Bio, Shiga, Japan). At 48-h post-transfection, the cells were rinsed twice with DPBS and lysed with 1.5 mL M-PER Mammalian Protein Extraction Reagent (Thermo Fisher Scientific) supplemented with a 1:200 dilution of protease inhibitor cocktail (set-III/EDTA-free) (Calbiochem, Merck KGaA, Darmstadt, Germany) and 25 U/mL Benzonase^®^ Nuclease (Novagen), and incubated at room temperature for 10 min with shaking. The preparation was centrifuged (15,000 × *g*, 4 °C, 5 min), and the protein content of the clear lysate was determined using the Quick Start Bradford Protein Assay (Bio-Rad Laboratories, Hercules, CA, USA). The presence of the Flag tag-fusion protein was verified by Western blot analysis using monoclonal anti-Flag M2 (Sigma-Aldrich, St. Louis, MO, USA), goat anti-mouse immunoglobulin-alkaline phosphatase (AP) conjugate (SouthernBiotech, Birmingham, AL, USA), and KPL BCIP/NBT substrate (SeraCare Life Sciences). For co-immunoprecipitation, cell lysates containing 10 µg of protein and 3 µg of individual R9-HuscFvs were added to the mixture in 1 mL of binding buffer (IBS containing 0.1% v/v Tween-20 and 0.01% w/v skim milk). A total of 200 μL of that mixture was added to an ANTI-FLAG^®^ M2-coated 96-well plate (Sigma-Aldrich), which was pre-blocked with Pierce Protein-Free Blocking Buffer (Thermo Fisher Scientific). The plate was kept at room temperature for 2 h on a rotating platform. After washing with wash buffer (IBS containing 0.1% v/v Tween-20), all wells were blocked with 240 μL of 1:1,000 biotin-blocking buffer (IBA) and kept at room temperature for 10 min. After removal of the blocking buffer, 200 μL of 1:4,000 Strep-Tactin^®^-HRP conjugate (IBA) was added, kept for an additional 1 h, and washed, and 200 µL of KPL ABTS substrate (SeraCare Life Sciences) was added. The OD_405nm_ of the content of each well was spectrometrically determined. The remaining portion of each mixture was subjected to Western blot analysis. Flag-tagged VP35 was detected using mouse monoclonal anti-Flag M2 (Sigma-Aldrich) and goat anti-mouse immunoglobulin-AP conjugate (SouthernBiotech). Strep tag II-tagged R9-HuscFvs were detected using Strep-Tactin^®^-AP conjugate (IBA Life Sciences, GmbH). BCIP/NBT substrate was used for color development.

### Biocompatibility of the transbodies

Culture medium (100 μL) containing 1 × 10^4^ HepG2 cells was placed in the wells of a 96-well white polystyrene microplate (Corning) and kept at 37 °C in a 5% CO_2_ incubator overnight. The culture medium was then replaced with 100 μL of fresh complete medium containing 25 µg/mL of transbodies (from titration) or storage buffer. Controls included cells in medium alone (minimal cytotoxicity/maximal cell viability), cells with added digitonin (maximal cytotoxicity), and complete medium without cells (luminescence background control). After leaving the microplate at 37 °C in a 5% CO_2_ atmosphere for 24 h, cytotoxicity was measured using a CytoTox-Glo Cytotoxicity Assay (Promega, Madison, WI, USA). Briefly, assay reagent (50 μL) was added to each well, mixed with the medium containing cells, and the mixture was kept at room temperature for 15 min before the luminescent signal was recorded with a Synergy™ H1 Hybrid Multi-Mode Microplate Reader (BioTek Instruments, Winooski, VT, USA).

### Construction of the EBOV minigenome reporter system

Plasmids for expressing Zaire EBOV NP, VP35, VP30, and L were designed as previously described^53^ with modifications. Optimized DNA coding for EBOV NP (accession MF801600), NP-IRES-VP35 (accession MF801600), VP30 (accession MF801601), and L (accession MF801602) was synthesized and cloned into the pCI-neo mammalian expression vector (with CMV promoter) (GenScript). The RNA polymerase I-driven transcription cassette for the synthesis of EBOV-like RNA (i.e., *EBOV leader-Gluc-EBOV trailer* (accession MF801603)) was generated in-house by splice overlapped extension PCR (SOE-PCR) using gBlocks® Gene Fragments (Integrated DNA Technologies) as a template. The cassette was cloned anti-directionally between the Pol I promoter and the terminator (Sal box) of pPol I (accession MF882921) using an In-Fusion HD Cloning Plus Kit (Takara Bio). The resulting construct, designated pPol-I-EBOV-Gluc, was introduced into *E. coli* JM110 and recovered using an EndoFree Plasmid Maxi Kit (Qiagen, Hilden, Germany). Plasmid cocktails used in the minigenome assay and in transbody-mediated inhibition of EBOV gene transcription and replication were prepared in endotoxin-free water, as follows: 5 µg/mL pCI-NP-IRES-VP35 or pCI-NP, 3 µg/mL pCI-VP30, 30 μg/mL pCI-L, and 2.5 μg/mL pPol-I-EBOV-Gluc.

### Inhibition of EBOV minigenome transcription by transbodies

Trypsinized COS-7 cells (8 × 10^5^ cells in 900 μL complete medium) were transfected by adding them to 100 μL of Xfect™ Single Shots (Midi) Transfection Reagent (Takara Bio) containing 4.05 μg of minigenome plasmid cocktail. The transfection mixture was added to cells in a sterile microcentrifuge tube and mixed gently. Immediately thereafter, 2 × 10^4^ cells in a 25-μL volume were dispensed into the individual wells of a 96-well cell culture cluster (Corning). Complete medium (100 μL) containing 25 µg/mL of R9-HuscFvs or antibody storage buffer was added to the transfected cells and incubated at 37 °C in a 5% CO_2_ incubator for 36 h. The *Gaussia* luciferase (Gluc) activities were measured using a Pierce *Gaussia* Luciferase Glow Assay Kit (Thermo Fisher Scientific) according to manufacturer’s instructions. Briefly, the culture medium was collected, and the cells were added to 100 µL of 1× lysis buffer with continuous shaking (400 rpm) at room temperature for 30 min. To obtain total luciferase activity expressed by the minigenome system, each cell lysate (10 µL) and cell culture medium (equal volume) were mixed and added to the appropriate well of a 96-well white polystyrene microplate (Corning). A bioluminescent reaction was generated by adding 50 μL of co-elenterazine solution to individual wells and kept at room temperature for 5 min. The luminescent signal of each well was recorded using a Synergy™ H1 Hybrid Multi-Mode Microplate Reader (BioTek Instruments).

### Generation of lentivector carrying EBOV *VP35*

Optimized DNA sequence coding for full-length VP35 of Zaire EBOV (accession MF801600) and mutant VP35 (H240E; codon CAC to GAG) were synthesized (GenScript) and subcloned into the pLVX-Puro vector backbone via the *Xho*I and *Bam*HI restriction sites. The recombinant constructs (pLVX-VP35 and pLVX-VP35H240E) were introduced into *E. coli* DH5α, and replicated plasmids were recovered using an EndoFree Plasmid Maxi Kit (Qiagen). To generate the VSV-G pseudotyped lentivector, lentiviral packaging was prepared by co-transfecting Lenti-X 293 -T cells with pLVX-VP35, pLVX-VP35H240E or empty vector in a Lenti-X™ HTX Packaging System (Integrase Deficient; Takara Bio). Lenti-X 293 -T cells (5 × 10^6^ cells) in 10 mL of tetracycline-free complete medium were placed in a 100-mm cell culture dish (Eppendorf) and kept overnight in a 37 °C CO_2_ incubator. DNA-Xfect Solution (Takara Bio) was added to the cells and incubated further for 4 h. After discarding the transfection medium, the cells were replenished with fresh medium containing tetracycline-free FBS. Viral protein expression and packing were allowed for 48 h in a 37 °C CO_2_ incubator. The presence of lentivectors in culture supernatants was checked using Lenti-X™ GoStix™ (Takara Bio), concentrated using a Lenti-X™ Concentrator (Takara Bio), and measured using a Lenti-X™ qRT-PCR Titration Kit (Takara Bio). The viral vector stock (adjusted to 10^8^–10^9^ copies/mL) was kept at −80 °C in single-use aliquots until use.

### Effect of VP35 transgene on the innate immune response of hepatic cells

HepG2 cells (3 × 10^5^ per well) in a 12-well cell culture cluster (Corning) were transduced with lentivector carrying the VP35 gene or an empty cassette at an MOI of 1.0 by spinoculation at 30 °C for 1 h. The cells were washed with DPBS, replenished with 1 mL fresh complete medium, and then incubated for 12 or 36 h. Control cells were replenished with fresh complete medium or medium containing 1 μg/mL poly(I:C)-HMW/LyoVec complexes (InvivoGen, San Diego, CA, USA). After the indicated incubation times, the culture medium was removed from each well; the cells were lysed with 0.4 mL of TRIzol® Reagent (Ambion, Thermo Fisher Scientific). Total RNA was extracted from the TRizol aqueous phase using the Total RNA Mini Kit (Geneaid Biotech). The RNA concentration was measured (NanoDrop 2000 Spectrophotometer, Thermo Fisher Scientific) and kept at −80 °C until use.

### Effect of transbodies on hepatic cells

HepG2 cells (3 × 10^5^ cells/well) were incubated with 1 mL of culture medium containing transbodies (25 μg/mL). Cells were kept in a 37 °C CO_2_ incubator for 12 h before total RNA extraction. Expression levels of the innate immune response genes were determined by quantitative reverse transcription-polymerase chain reaction (qRT-PCR) (Supplementary Method S3).

### IFN-β responses of the transbody-treated *VP35*-transduced cells

HepG2 cells (3 × 10^5^ cells/well) were transduced with lentivector carrying the EBOV VP35 gene cassette at an MOI of 1.0. The cells were washed with DPBS, replenished with 1 mL of fresh medium, and incubated for 24 h. The spent medium was discarded, and 1 mL of fresh medium containing 25 μg/mL of transbodies or 1 μg/mL poly(I:C)-HMW/LyoVec complexes was added to the cells. After 12 h, total RNA was extracted, and the expression levels of host innate immune response genes (*IFNB1* coding for IFN-β and *EIF2AK2* coding for PKR) were determined by qRT-PCR.

### Statistical analysis

GraphPad Prism version 6 (La Jolla, CA, USA) was used to compare the results (mean ± standard deviation) of all tests. Statistical significance was determined using the tests indicated in the figure legends. A *p*-value < 0.05 was statistically significant.

## Electronic supplementary material

Supplementary Method S1

Supplementary Method S2

Supplementary Method S3

Supplementary Figure S1

Supplementary Figure S2

Supplementary Table S1

## References

[1] Leung DW, Prins KC, Basler CF, Amarasinghe GK (2010). Ebolavirus VP35 is a multifunctional virulence factor. Virulence.

[2] Mühlberger E, Lötfering B, Klenk HD, Becker S (1998). Three of the four nucleocapsid proteins of Marburg virus, NP, VP35, and L, are sufficient to mediate replication and transcription of Marburg virus-specific monocistronic minigenomes. J. Virol..

[3] Noda T, Aoyama K, Sagara H, Kida H, Kawaoka Y (2005). Nucleocapsid-like structures of Ebola virus reconstructed using electron tomography. J. Vet. Med. Sci..

[4] Basler CF (2000). The Ebola virus VP35 protein functions as a type I IFN antagonist. Proc. Natl Acad. Sci. USA.

[5] Basler CF, García-Sastre A (2002). Viruses and the type I interferon antiviral system: induction and evasion. Int. Rev. Immunol..

[6] Basler CF (2003). The Ebola virus VP35 protein inhibits activation of interferon regulatory factor 3. J. Virol..

[7] Basler CF, Amarasinghe GK (2009). Evasion of interferon responses by Ebola and Marburg viruses. J. Interferon Cytokine Res..

[8] Cárdenas WB (2006). Ebola virus VP35 protein binds double-stranded RNA and inhibits alpha/beta interferon production induced by RIG-I signaling. J. Virol..

[9] Yen B, Mulder LC, Martinez O, Basler CF (2014). Molecular basis for ebolavirus VP35 suppression of human dendritic cell maturation. J. Virol..

[10] Prins KC, Cárdenas WB, Basler CF (2009). Ebola virus protein VP35 impairs the function of interferon regulatory factor-activating kinases IKK-epsilon and TBK-1. J. Virol..

[11] Haller O, Kochs G, Weber F (2006). The interferon response circuit: induction and suppression by pathogenic viruses. Virology.

[12] Hartman AL, Towner JS, Nichol ST (2004). A C-terminal basic amino acid motif of Zaire ebolavirus VP35 is essential for type I interferon antagonism and displays high identity with the RNA-binding domain of another interferon antagonist, the NS1 protein of influenza A virus. Virology.

[13] Leung DW (2009). Structure of the Ebola VP35 interferon inhibitory domain. Proc. Natl Acad. Sci. USA.

[14] Kimberlin CR (2010). Ebolavirus VP35 uses a bimodal strategy to bind dsRNA for innate immune suppression. Proc. Natl Acad. Sci. USA.

[15] Reid SP, Cárdenas WB, Basler CF (2005). Homo-oligomerization facilitates the interferon-antagonist activity of the ebolavirus VP35 protein. Virology.

[16] Leung DW (2015). An intrinsically disordered peptide from Ebola virus VP35 controls viral RNA synthesis by modulating nucleoprotein-RNA interactions. Cell Rep..

[17] Kirchdoerfer RN, Abelson DM, Li S, Wood MR, Saphire EO (2015). Assembly of the Ebola virus nucleoprotein from a chaperoned VP35 complex. Cell Rep..

[18] Luthra P, Jordan DS, Leung DW, Amarasinghe GK, Basler CF (2015). Ebola virus VP35 interaction with dynein LC8 regulates viral RNA synthesis. J. Virol..

[19] Prins KC (2010). Basic residues within the ebolavirus VP35 protein are required for its viral polymerase cofactor function. J. Virol..

[20] Leung DW (2010). Structural basis for dsRNA recognition and interferon antagonism by Ebola VP35. Nat. Struct. Mol. Biol..

[21] Clark DV, Jahrling PB, Lawler JV (2012). Clinical management of Filovirus-infected patients. Viruses.

[22] Geisbert TW (2003). Treatment of Ebola virus infection with a recombinant inhibitor of factor VIIa/tissue factor: a study in rhesus monkeys. Lancet.

[23] Warfield KL (2006). Gene-specific countermeasures against Ebola virus based on antisense phosphorodiamidate morpholino oligomers. PLoS Pathog..

[24] Hensley LE (2007). Recombinant human activated protein C for the postexposure treatment of Ebola hemorrhagic fever. J. Infect. Dis..

[25] Geisbert TW (2010). Postexposure protection of non-human primates against a lethal Ebola virus challenge with RNA interference: a proof-of-concept study. Lancet.

[26] Warren TK (2014). Protection against filovirus diseases by a novel broad-spectrum nucleoside analogue BCX4430. Nature.

[27] Picazo E, Giordanetto F (2015). Small molecule inhibitors of ebola virus infection. Drug Discov. Today.

[28] Kilgore PE, Grabenstein JD, Salim AM, Rybak M (2015). Treatment of ebola virus disease. Pharmacotherapy.

[29] Henao-Restrepo AM (2015). Efficacy and effectiveness of an rVSV-vectored vaccine expressing Ebola surface glycoprotein: interim results from the Guinea ring vaccination cluster-randomised trial. Lancet.

[30] Jahrling PB, Geisbert JB, Swearengen JR, Larsen T, Geisbert TW (2007). Ebola hemorrhagic fever: evaluation of passive immunotherapy in nonhuman primates. J. Infect. Dis..

[31] Maruyama T (1999). Ebola virus can be effectively neutralized by antibody produced in natural human infection. J. Virol..

[32] Parren PW, Geisbert TW, Maruyama T, Jahrling PB, Burton DR (2002). Pre- and postexposure prophylaxis of Ebola virus infection in an animal model by passive transfer of a neutralizing human antibody. J. Virol..

[33] Oswald WB (2007). Neutralizing antibody fails to impact the course of Ebola virus infection in monkeys. PLoS Pathog..

[34] Marzi A (2012). Protective efficacy of neutralizing monoclonal antibodies in a nonhuman primate model of Ebola hemorrhagic fever. PLoS ONE.

[35] Qiu X (2012). Successful treatment of ebola virus-infected cynomolgus macaques with monoclonal antibodies. Sci. Transl. Med..

[36] Qiu X (2014). Reversion of advanced Ebola virus disease in nonhuman primates with ZMapp. Nature.

[37] Olinger GG (2012). Delayed treatment of Ebola virus infection with plant-derived monoclonal antibodies provides protection in rhesus macaques. Proc. Natl Acad. Sci. USA.

[38] Pettitt J (2013). Therapeutic intervention of Ebola virus infection in rhesus macaques with the MB-003 monoclonal antibody cocktail. Sci. Transl. Med..

[39] de La Vega MA, Wong G, Kobinger GP, Qiu X (2015). The multiple roles of sGP in Ebola pathogenesis. Viral Immunol..

[40] Corti D (2016). Protective monotherapy against lethal Ebola virus infection by a potently neutralizing antibody. Science.

[41] Forthal DN (2014). Functions of antibodies. Microbiol. Spectr..

[42] Kristensen M, Birch D, Mørck Nielsen H (2016). Applications and challenges for use of cell-penetrating peptides as delivery vectors for peptide and protein cargos. Int. J. Mol. Sci..

[43] Liu J (2014). Cell-penetrating peptide-mediated delivery of TALEN proteins via bioconjugation for genome engineering. PLoS ONE.

[44] Kulkeaw K (2009). Human monoclonal ScFv neutralize lethal Thai cobra, *Naja kaouthia*, neurotoxin. J. Proteom..

[45] Luthra P, Sun D, Silverman RH, He B (2011). Activation of IFN-β expression by a viral mRNA through RNase L and MDA5. Proc. Natl Acad. Sci. USA.

[46] Escudero-Pérez B, Volchkova VA, Dolnik O, Lawrence P, Volchkov VE (2014). Shed GP of Ebola virus triggers immune activation and increased vascular permeability. PLoS. Pathog..

[47] Rivera A, Messaoudi I (2015). Pathophysiology of Ebola virus infection: current challenges and future hopes. ACS Infect. Dis..

[48] Darling TL, Sherwood LJ, Hayhurst A (2017). Intracellular cross-linking of filoviral nucleoproteins with Xintrabodies restricts viral packaging. Front. Immunol..

[49] Garza JA, Taylor AB, Sherwood LJ, Hart PJ, Hayhurst A (2017). Unveiling a drift resistant cryptotope within Marburgvirus nucleoprotein recognized by llama single-domain antibodies. Front. Immunol..

[50] Kugelman JR (2015). Emergence of Ebola virus escape variants in infected nonhuman primates treated with the MB-003 antibody cocktail. Cell Rep..

[51] Jasenosky LD, Neumann G, Kawaoka Y (2010). Minigenome-based reporter system suitable for high-throughput screening of compounds able to inhibit Ebolavirus replication and/or transcription. Antimicrob. Agents Chemother..

[52] Edwards MR (2015). High-throughput minigenome system for identifying small-molecule inhibitors of Ebola virus replication. ACS Infect. Dis..

[53] Zhang YJ, Ding JN, Zhong H, Han JG (2017). Exploration micromechanism of VP35 IID interaction and recognition dsRNA: A molecular dynamics simulation. Proteins.

[54] The UniProt Consortium. (2017). UniProt: the universal protein knowledgebase. Nucleic Acids Res..

[55] Retter I, Althaus HH, Münch R, Müller W (2005). VBASE2, an integrative V gene database. Nucleic Acids Res..

[56] Roy A, Kucukural A, Zhang Y (2010). The Protein Data Bank. Nucleic Acids Res..

[57] Kozakov D (2017). Improving the physical realism and structural accuracy of protein models by a two-step atomic-level energy minimization. Biophys. J..

[58] Hospital A (2012). Atomic-level protein structure refinement using fragment-guided molecular dynamics conformation sampling. Structure.

