# Supplementary material for: Human transbodies that interfere with the functions of Ebola virus VP35 protein in genome replication and transcription and innate immune antagonism
Source: Emerg Microbes Infect. 2018 Mar 21;7:41. doi: 10.1038/s41426-018-0031-3 (PMC5864874; doi:10.1038/s41426-018-0031-3)
Supplement: Supplementary file 1 — Supplementary Method S1 [file 41426_2018_31_MOESM1_ESM.docx]

**Supplementary Method S1** Purification of bacterial inclusion bodies and recombinant protein refolding. For isolation and purification of the bacterial inclusion bodies (IBs), 2 grams of transformed *E. coli* wet pellet were lysed with 10 mL of BugBuster^®^ Protein Extraction Reagent (Novagen, Merck KGaA, Darmstadt, Germany) supplemented with 20 μL of Lysonase™ Bioprocessing Reagent (Novagen). Preparations were kept at 25 °C with low speed shaking for 20 min. IB pellets were harvested by centrifugation at 8,000 × *g* at 4 °C for 30 min; washed twice with 20 mL of Wash-100 buffer [50 mM sodium phosphate buffer, pH 8.0; 500 mM NaCl; 5 mM EDTA; 8% w/v glycerol and 1% v/v Triton X-100] at 25 °C for 40 min; washed once with Wash-114 buffer [50 mM Tris buffer, pH 8.0; 300 mM NaCl and 1% v/v Triton X-114] at 4 °C for 20 min; and, washed once with Wash-Solvent buffer [50 mM Tris buffer, pH 8.0 and 60% v/v isopropanol] at 25 °C for 20 min by shaking the preparation in the wash fluids at high speed, followed by centrifugation. The preparations were supplemented with 20 mL of Milli-Q^®^ water (Merck Millipore), placed on a shaker at 25 °C for 20 min, and centrifuged at 8,000 × *g* for 20 min. IBs were suspended in ultrapure water and stored at 4 °C. For protein refolding, IBs were solubilized at 1 mg/mL in solubilization buffer [50 mM CAPS, pH 11.0; 0.3% w/v N-lauryl sarcosine and 1 mM DTT] and kept at 4 °C overnight. After being completely dissolved, the protein was loaded into Slide-A-Lyzer G2 Dialysis Cassettes (K2) (Thermo Fisher Scientific) and dialyzed at 4 °C with slow stirring against 750 mL refolding buffer [20 mM Tris, pH 8.5 for bVP35FL and bVP35IID preparation and 20 mM imidazole, pH 8.5 supplemented with 0.1 mM DTT for R9-HuscFv preparation]. After 3 h, the preparation was dialyzed in fresh refolding buffer at 4 °C overnight. The refolded protein was subsequently dialyzed at 4 °C for 3 h with slow stirring against dialysis buffer (20 mM Tris, pH 8.5 for bVP35FL and bVP35IID and 20 mM imidazole, pH 8.5 for R9-HuscFvs) at 4 °C overnight. The protein was then filtered through a 0.2 µm low protein binding Acrodisc^®^ syringe filter (Pall, Port Washington, NY, USA) and kept in a 30 °C water-bath for an additional 2 h before being supplemented with 8% w/v glycerol for rVP35FL and bVP35IID and 60 mM trehalose for R9-HuscFvs. Protein concentrations were measured using a Pierce BCA™ (bicinchoninic acid) Protein Assay (Thermo Fisher Scientific). Quality and purity of proteins were determined by SDS-PAGE. Binding activity of refolded R9-HuscFvs to bVP35FL and bVP35IID, and their cell penetrable activity were determined by indirect ELISA and confocal microscopy (Carl Zeiss Laser Scanning System LSM 510, Carl Zeiss Microscopy GmbH, Jena, Germany), respectively. All preparations were stored at -80 °C until use.
