# Supplementary material for: Human transbodies that interfere with the functions of Ebola virus VP35 protein in genome replication and transcription and innate immune antagonism
Source: Emerg Microbes Infect. 2018 Mar 21;7:41. doi: 10.1038/s41426-018-0031-3 (PMC5864874; doi:10.1038/s41426-018-0031-3)
Supplement: Supplementary file 2 — Supplementary Method S2 [file 41426_2018_31_MOESM2_ESM.docx]

**Supplementary Method S2** Indirect ELISA for determining R9-HuscFv binding activity and half maximal effective concentration (EC_50_). Wells of a 96-well EIA/RIA plate (Corning) were individually coated with 50 nM of bVP35FL, bVP35IID, or BSA antigen control in 100 μL of coating buffer, and the plate was kept at 4 °C overnight. Unbound materials were washed away with PBS and the empty sites on the well surface were blocked with 200 μL of blocking buffer [5% w/v skim milk in imidazole buffered saline (IBS)] at 37 °C for 1 h. After removing the excess blocking buffer, excess purified refolded R9-HuscFv (over antigen) in 100 µL IBS containing 0.05% Tween-20 (IBST) and blank (IBST alone) were added to the wells. The plate was kept at 25 °C on a rotator for 1 h and then washed. One hundred and fifty μL of 1:1,000 biotin blocking buffer (IBA Life Sciences GmbH, Göttingen, Germany) in IBST was added and the plate was kept at 25 °C for 10 min. The blocking buffer was then discarded. One hundred µL of 1:4,000 Strep-Tactin^®^-HRP conjugate (IBA Life Sciences GmbH) in IBST was added to each well and the plate was kept at 25 °C for 1 h on a rotator. After washing, 100 µL of ABTS peroxidase substrate (KPL, SeraCare Life Sciences, Milford, MA, USA) was added to individual wells. After incubation, the optical density (OD_405nm_) of the content in each well was determined against blank. Positive binding of R9-HuscFvs to VP35 proteins was OD_405nm_ ≥ 3× higher than the OD_405nm_ to the BSA control.

For determining the EC_50_ of the R9-HuscFvs, direct-binding ELISA was performed. Briefly, 100 μL of bVP35FL (50 nM in coating buffer), was coated into individual wells of a 96-well EIA/RIA plate (Corning) and the plate was kept at 4 °C overnight. The wells were blocked with 200 μL of blocking buffer as above. After discarded the blocking buffer, 100 µL of serial concentrations of purified refolded R9-HuscFvs in IBST (10 μM to 100 pM) were added to the wells and kept at 25 °C for 1 h. Wells added with IBST served as blank. After washing, biotin blocking buffer (IBA Life Sciences GmbH) (200 μL of 1:1,000) was added to each well, kept at 25 °C for 10 min and the fluids were discarded. The plate was incubated with Strep-Tactin^®^-HRP conjugate (IBA Life Sciences GmbH) (100 μL of 1:4,000) for 1 h. ABTS peroxidase substrate (KPL) was used as a substrate. Optical density (OD_405nm_) of the content in each well was determined against blank. The EC_50_ of each transbody was generated by using EC50 Calculator (https://www.aatbio.com/tools/ec50-calculator).
