# Supplementary material for: Human transbodies that interfere with the functions of Ebola virus VP35 protein in genome replication and transcription and innate immune antagonism
Source: Emerg Microbes Infect. 2018 Mar 21;7:41. doi: 10.1038/s41426-018-0031-3 (PMC5864874; doi:10.1038/s41426-018-0031-3)
Supplement: Supplementary file 3 — Supplementary Method S3 [file 41426_2018_31_MOESM3_ESM.docx]

**Supplementary Method S3** Quantitative Reverse Transcription-PCR (qRT-PCR)**.** The primers used in this study were obtained from a public resource for PCR primers^1^ (PrimerBank, http://pga.mgh.harvard.edu/primerbank), as follows:

ID 50593016c1; amplicon size 88 bp

*IFNB1* forward 5΄-ATGACCAACAAGTGTCTCCTCC-3΄

*IFNB1* reverse 5΄-GGAATCCAAGCAAGTTGTAGCTC-3΄

ID 378404907c3; amplicon size 92 bp

*EIF2AK2* forward 5΄-GCCGCTAAACTTGCATATCTTCA-3΄

*EIF2AK2* reverse 5΄-TCACACGTAGTAGCAAAAGAACC-3΄

ID 378404907c3; amplicon size 101 bp

*GAPDH* forward 5΄-CTGGGCTACACTGAGCACC-3΄

*GAPDH* reverse 5΄-AAGTGGTCGTTGAGGGCAATG-3΄

Each reaction mixture (12.5 μL volume) contained 6.25 μL 2× Brilliant II SYBR Green QRT-PCR Master Mix (Agilent Technologies, Santa Clara, CA, USA), 400 nM final concentration of each forward and reverse primer, 0.5 μL RT/RNase block enzyme mixture, and 200 ng RNA template in nuclease-free PCR-grade water. The reaction was performed in an Mx3000P QPCR System (Agilent Technologies). The following thermal cycles were used: reverse transcription at 42 °C for 30 min and 55 °C for 30 min, followed by initial denaturation at 95 °C for 10 min and 45 cycles at 95 °C for 30 s, 60 °C for 30 s, and 72 °C for 30 s. A dissociation curve was generated from a thermal profile consisting of 95 °C for 1 min, 55 °C for 45 s, and 95 °C (0.5 °C/s). Each sample was amplified in triplicate. Levels of *GAPDH* transcripts were used for normalization. Gene expressions relative to normal cells were analyzed using the relative quantity (2^-ΔΔCt^) method.

**Reference**

1. Spandidos A, Wang X, Wang H, Seed B. PrimerBank: a resource of human and mouse PCR primer pairs for gene expression detection and quantification. *Nucleic Acids Res* 2010; **38**: D792−D799.
