# Supplementary material for: Human transbodies that interfere with the functions of Ebola virus VP35 protein in genome replication and transcription and innate immune antagonism
Source: Emerg Microbes Infect. 2018 Mar 21;7:41. doi: 10.1038/s41426-018-0031-3 (PMC5864874; doi:10.1038/s41426-018-0031-3)

**Supplementary Figure S1** Screening of *E. coli* clones that produced bVP35FL-bound HuscFvs by indirect ELISA. Purified bVP35FL and BSA were used as test and control antigens, respectively. *E. coli* HB2151 homogenates containing soluble HuscFvs (clones 1 to 51) were incubated with the test antigen in wells side-by-side with the control antigen. Positive binding was determined by the OD_405nm_ signal above mean + 3 SD of the background binding control (lysate of original *E. coli* HB2151; HB).


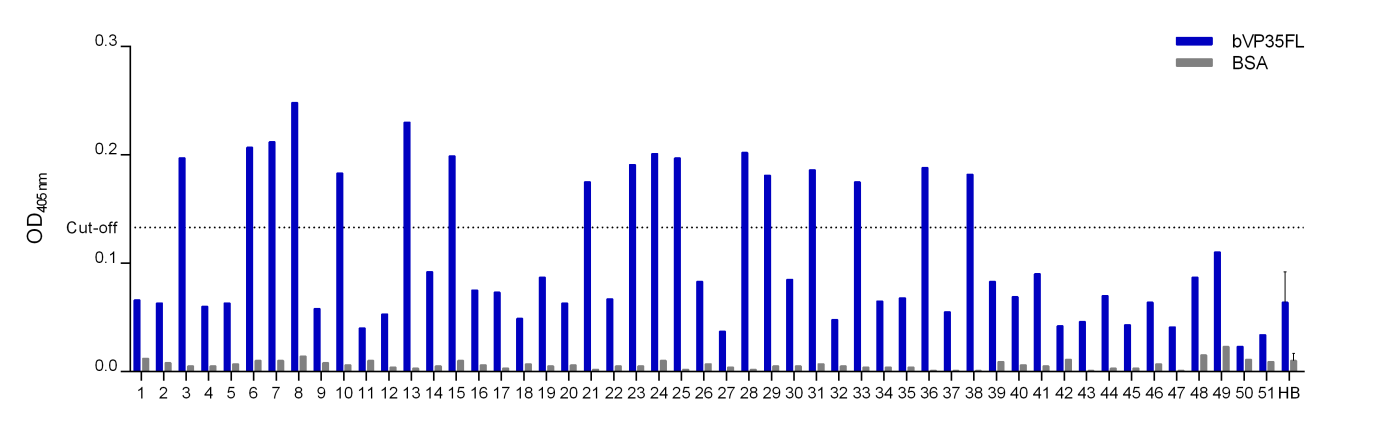

Supplement: Supplementary file 4 — Supplementary Figure S1 [file 41426_2018_31_MOESM4_ESM.docx]
