# Supplementary material for: Human transbodies that interfere with the functions of Ebola virus VP35 protein in genome replication and transcription and innate immune antagonism
Source: Emerg Microbes Infect. 2018 Mar 21;7:41. doi: 10.1038/s41426-018-0031-3 (PMC5864874; doi:10.1038/s41426-018-0031-3)

**Supplementary Figure S2** EC_50_ of R9-HuscFvs that bound to VP35-IID as determined by direct-binding ELISA. Serial concentrations of purified, refolded R9-HuscFvs at 10 μM to 100 pM were incubated with immobilized bVP35FL (50 nM) in EIA/RIA wells. OD_405nm_ signals were determined against blank (antigen coated well incubated with diluent). The EC_50_ graphs of (A) R9-HuscFv3, (B) R9-HuscFv8, (C) R9-HuscFv13, and (D) R9-HuscFv24 were generated using EC50 Calculator. The molecular weights of the R9-HuscFvs were computed by using ProtParam (http://web.expasy.org/protparam).


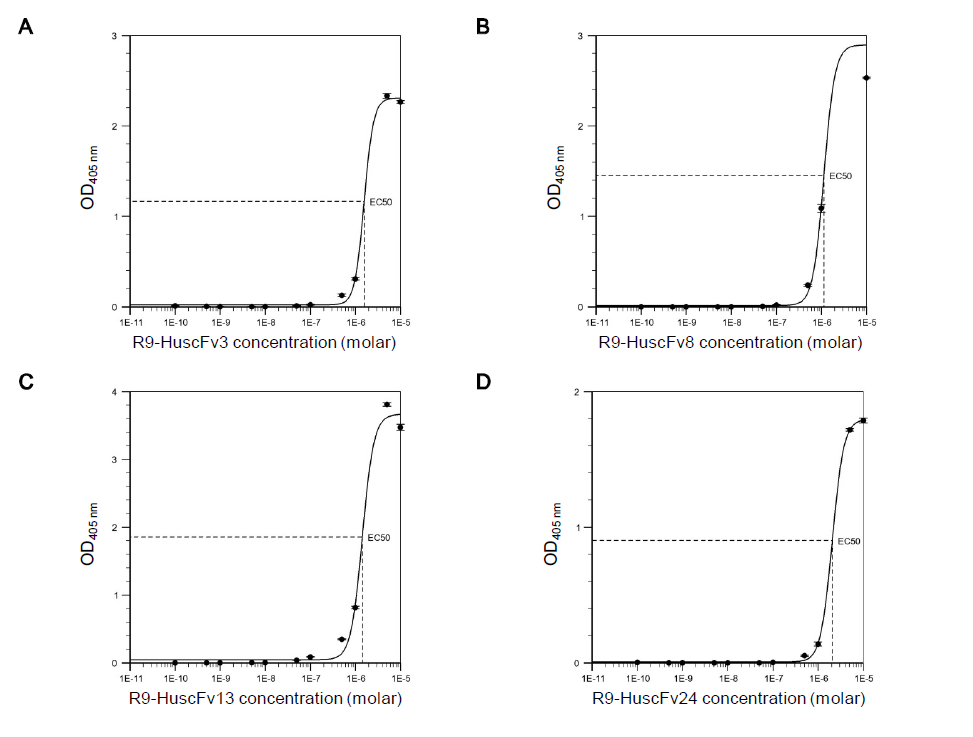

Supplement: Supplementary file 5 — Supplementary Figure S2 [file 41426_2018_31_MOESM5_ESM.docx]
