# Supplementary material for: Human transbodies that interfere with the functions of Ebola virus VP35 protein in genome replication and transcription and innate immune antagonism
Source: Emerg Microbes Infect. 2018 Mar 21;7:41. doi: 10.1038/s41426-018-0031-3 (PMC5864874; doi:10.1038/s41426-018-0031-3)
Supplement: Supplementary file 6 — Supplementary Table S1 [file 41426_2018_31_MOESM6_ESM.docx]

**Supplementary Table S1** Contact residues between VP35-IID and HuscFvs identified by antibody-protein docking and simulation

| **Interface binding of HuscFv3** | |  | **Interface binding of HuscFv8** | |  | **Interface binding of HuscFv13** | |  | **Interface binding of HuscFv24** | |
| --- | --- | --- | --- | --- | --- | --- | --- | --- | --- | --- |
| **VP35IID** | **Function** |  | **VP35IID** | **Function** |  | **VP35IID** | **Function** |  | **VP35IID** | **Function** |
| **P233** | dsRNA contact |  | **E269** | IID multimeric contact |  | **M214** | Unknown |  | **P217** | Unknown |
| **F235** | End-cap recognition |  | **G270** | IID multimeric contact |  | **G215** | Unknown |  | **D218** | Unknown |
| **T237** | dsRNA contact |  | **D271** | IID multimeric contact |  | **P217** | Unknown |  | **I219** | Unknown |
| **A238** | Unknown |  | **S272** | dsRNA contact |  | **D218** | Unknown |  | **S220** | Unknown |
| **F239** | End-capped dsRNA |  | **C275** | dsRNA contact |  | **I219** | Unknown |  | **A221** | Unknown |
| **D271** | IID dimer contact |  | **I278** | dsRNA contact |  | **S220** | Unknown |  | **K222** | First basic patch |
| **S272** | dsRNA contact |  | **Q279** | dsRNA contact |  | **A221** | Unknown |  | **D223** | Unknown |
| **P273** | Unknown |  | **K282** | Border basic residue |  | **K222** | First basic patch |  | **R225** | First basic patch |
| **Q274** | dsRNA contact |  | **R283** | Border basic residue |  | **D223** | Unknown |  | **K248** | First basic patch |
| **C275** | dsRNA contact |  | **Q288** | Unknown |  | **R225** | First basic patch |  | **L249** | Unknown |
| **I278** | dsRNA contact |  | **D289** | Unknown |  | **N226** | Unknown |  | **K251** | First basic patch |
| **K282** | Border basic residue |  | **P315** | Unknown |  | **Y229** | Unknown |  | **D252** | Unknown |
| **R300** | Border basic residue |  | **S317** | Unknown |  | **D230** | Unknown |  | **S253** | Unknown |
| **R305** | Central basic patch |  | **P318** | Unknown |  | **L232** | Unknown |  | **N254** | Unknown |
| **A306** | dsRNA contact |  | **D321** | Unknown |  | **G234** | Unknown |  | **S255** | Unknown |
| **Q308** | Unknown |  | **R322** | Central basic patch |  | **F235** | End-cap recognition |  | **L256** | Unknown |
| **K309** | Central basic patch |  | **G323** | Unknown |  | **G236** | Unknown |  | **D257** | Unknown |
| **S310** | dsRNA contact |  | **W324** | Unknown |  | **H240** | End-cap recognition |  | **I258** | Unknown |
| **R312** | Central basic patch |  | **K339** | Central basic patch |  | **Q244** | Unknown |  | **P285** | Unknown |
| **P313** | Unknown |  | **I340** | End-capped dsRNA |  | **K248** | First basic patch |  | **I286** | Unknown |
| **P315** | Unknown |  |  |  |  | **K251** | First basic patch |  |  |  |
| **R322** | Central basic patch |  |  |  |  | **D252** | Unknown |  |  |  |
| **G323** | Unknown |  |  |  |  | **N254** | Unknown |  |  |  |
| **W324** | Unknown |  |  |  |  | **L256** | Unknown |  |  |  |
| **K339** | Central basic patch |  |  |  |  |  |  |  |  |  |
| **I340** | End-capped dsRNA |  |  |  |  |  |  |  |  |  |

VP35-IID functions according to references 19 and 20

Interactive residues of VP35 that interacted directly with HuscFvs by hydrogen bond, π-effects, and/or hydrophobic interaction are indicated in red

Interactive residues of VP35 in contact interface that fell within a 5 Å threshold of van der Waals radii of HuscFv are indicated in black
